# Supplementary material for: Impact of Starch Binding Domain Fusion on Activities and Starch Product Structure of 4-α-Glucanotransferase
Source: Molecules. 2023 Jan 30;28(3):1320. doi: 10.3390/molecules28031320 (PMC9920598; doi:10.3390/molecules28031320)
Supplement: Supplementary file 1 [file molecules-28-01320-s001.zip › molecules-2182328-supplementary.pdf]

Figure S1. Sequence alignment of CBM20s with focus on GH77 DPE2s. The alignment of all 87 CBM20 domains from 65 selected enzymes having the CBM20 (Table 1). The most important positions involved in starch-binding sites 1 and 2 – recognized in the CBM20s from *Aspergillus niger* GH15 glucoamylase (site 1: Trp543, Lys578 and Trp590; site 2: Tyr527, Tyr556 and Trp563) [9] and *Bacillus circulans* strain 251 GH13_2 cyclodextrin glucanotransferase (site 1: Trp616, Lys651 and Trp662; site 2: Tyr633 and Trp636) [13] – are indicated, respectively, above and below the alignment by numbers “1” and “2”. If conserved, the sites 1 and 2 are highlighted in yellow and blue, respectively; conserved and non-conserved substitutions being coloured red and gray, respectively. The labels of protein sources consist of the name of the organism, letter “A”, “B” or “E” for the archaeal, bacterial and eukaryotic origin, respectively, CAZy family affiliation (if any), enzyme abbreviated name (for details, see Table 1) and the UniProt accession number. If there are more CBM20 copies for a single protein, the copies in the order of their appearance in the sequence are also indicated by the relevant number “1”, “2” and “3” (at the end of the protein label). The three CBM20 domains studied in the present work – two from GH77 *Solanum tuberosum* DPE2 and one from GH15 *Aspergillus niger* glucoamylase – are marked by an asterisk. The sequence order in the alignment (starting from the top) reflects their order in the tree in the anticlockwise manner (starting from the first sequence in the eukaryotic DPE2 cluster of CBM20 copies 2).

```
                                                                                       2            1                  2     2                    1                   1
Alistipes_finegoldii_B_GH77_DPE2_I3YMP0_2                 ---NPKKAAGNITLRVIL-PT----LRPDETLAVAGSGRELGDWK--RIVPMD-DS--------------RFPEWELTL---HTAH------------------RFEYKFLIADRK-TLTP-------------ILWEEGANRTWGELPGAG------EHALDAAAYPRFPERRWQG--------  111
Barnesiella_intestinihominis_B_GH77_DPE2_K0XAQ2_2         ---VETIEPGTLTVKVDA-SQ----LRPDERLVMVGDCPELGDWDVLKAPLLN-DS--------------AFPEWQITL---DGKQL--------------K-F-PFEYKFVIVRTH-QREV-------------VAWETGENRICREKPQNPS-----EAVVVSGLRFNGSESQWRG--------  117
Tannerella_sp_CT1_B_GH77_DPE2_G9S294_2                    ---VLPSYSTAVTLQIYA-PS----IRPDEVVALTGTMM-SEAWSIKDAIIMD-DT--------------RFPLWEAVL---DASSL--------------K-H-TVEYKFFLVMKD-SRNV-------------VAWEGSNNRVLEIPLGPG-----ENIVFGSFKWNL--------------  109
Prevotella_denticola_B_GH77_DPE2_F2KWM4_2                 ---RLNNYNKAVTLKVRA-PQ----LGAADELYLTGAEPALGAWNVKKALKMV-QH--------------NINEWTLTL---DAAKL--------------S-GDQMEIKFFARSHD-DDAS-------------PVWEYSNNRTLELPAMEEG-----ETIVYELDE----------------  108
Dysgonomonas_mossii_B_GH77_DPE2_F8WZF9_2                  ---KLKYKKTILLKVNC-PY----IKKNQTMILSGSSKGLGEWNIEKAIHLT-PA--------------NFGEWQVVI---NATSV--------------K-T-PQEYKLAIYDNS-EKKI-------------IHWEKGSNRVLSPITEKED-----TVR-VEALDYRH---EWMS-------  113
Tannerella_forsythia_B_GH77_DPE2_G8UKR6_2                 ---IHEQPDRSLIIRVPN-PR----VRKNQRVALAGNQACLGHWDTTQAKLLC-PS--------------GAAEWEVCL---NADEI--------------S-Y-PFEYKFFVCDET-KRV--------------CHWEKGDNRVLAKTPSATC-----EVTVISEYPYREDLPEWKG--------  116
Parabacteroides_distasonis_B_GH77_DPE2_A6L7Y4_2           ---RVVRSGRKLVIKISA-PR----VEKNQCVAITGNQECLGNWHPDKALLS-CD--------------TFPEWHIDL---DAAEI--------------R-Y-PLEYKFLVWDND-SRQP-------------LYWESGENRILSLVPQKQD-----ETVVISGLYFRDSLPWRC--------  117
Bacteroides_thetaiotaomicron_B_GH77_DPE2_Q8A5U2_2         ---IPPCHRKGLVIKAYA-PR----INKDYCLAICGNQKALGNWDPDKAIPMS-DA--------------NFPEWQIEL---DASKL--------------K-F-PLEYKFLILYHKE-EKKA-------------DCWENNPNRYLADPEKLKTN----ETLVISDRYAYFDIPVWKG--------  117
Micromonas_sp_RCC299_E_GH77_DPE2_C1FJ00_2                 THSRDFIH---IRLEVRT-LR----LLPGQRLTATGDCAALGGWERDAAPVMNLDA---------------VTRTWEARV---DVAPE--------------D-L-PVRIVARDGAT-RAA--------------DAMEKGDVRVAPLGWIGE---SPSRIVARDGHFRYPG-AWRG-------  118
Trichomonas_vaginalis_E_GH77_DPE2_A2D7I8_2                KQTGDAIQ---TYFATNC-PY----VRPNQVLRIVGNVPELGNWDPKNSLVLA-DG--------------CFPAWAGNK---VFAKS--------------S-F-PFEYKYVIENKD-NST--------------FIWEECENRVCPVPVLSDG----KV-I---NYIQ-DW-FV----------  108
Polysphondylium_pallidum_E_GH77_DPE2_D3B4Z9_2             PSSPDKVV---IHFQVRC-TN----VQPSYNVYLVGSNMTLGNWRTERAIKLS-DQ--------------DFPIWKADL---EFTKD--------------Q-L-PFSYKYIIADKS-KTY--------------IHWEQGNDRWFTSSILPAS-----EY-SNTRSEER-DY-YFND--------  113
Dictyostelium_discoideum_E_GH77_DPE2_Q54PW3_2             QTQADKVI---IHFQVKS-TY----VPPNHSIYIVGSNTQLGNWRTDRAVKLS-DE--------------SYPIWKVDL---EFSKD--------------Q-L-PFSYKYIIKKD-KSV--------------VHWEYGNDRWFTASATHAS-----EY-SSTTNEDR-D-------------  108
Chlamydomonas_reinhardtii_E_GH77_DPE2_A8JEI0_2            SPAPGETV---VRLQVAD-FQ----LEDGEAVLVTGGIPQLGNWQPDQMCQLT-ET--------------HTPFWEAEL---RVPYS--------------C-F-PFTYKYAIQTA---EGL------------VAGRVCAVQRQVDVAVQSSSEVMSGVRAARGAAR-DR-HWRG-------  117
Volvox_carteri_E_GH77_DPE2_D8UDU0_2                       APAPGETV---LRLAVAD-FQ----LDAAEVLLVTGGISQLGNWQPDQMLRLT-ET--------------HTPFWEAEL---RVPYS--------------C-F-PFTYKYAIQTA---DGL------------VLEVGEPRLASLPSS-ASVGAPTVMVRHDGYFRR-DR-HWRG-------  116
*Solanum_tuberosum_E_GH77_DPE2_Q6R608_2                   LDQDGSVI---LQFRICC-PY----LEEGTSIYVLGSSLNLGQWKIQDGLKLV-YA--------------GDSLWQAAC---VMGKD--------------D-F-PLKYKYCKHSK---AGT------------SVECGASREISVDVT-TG---ESRFVVLSDGLMR-EM-PWRG-------  113
Populus_trichocarpa_E_GH77_DPE2_B9IHJ8_2                  KEGLDAVV---VHFKICC-PD----VEEETSVYVIGSTAKLGQWKVQDGLKLN-YA--------------GDSVWQAGA---LMQKG--------------D-F-PIKYKYCKYGK---AGN------------FSLETGAHRDLSIDSS-KV---PPRYIFLSDGMMR-EM-PWRG-------  114
Arabidopsis_thaliana_E_GH77_DPE2_Q8RXD9_2                 SDQDDSVV---VQFKICC-PD----IGEGTSVYVLGTPEKLGNWKVENGLRLN-YV--------------DDSIWEADC---LIPKA--------------D-F-PIKYRYCKVQK---EDS------------IGFESGGNRELSLHSI-GS---KQEYIVMSDGLFR-AM-PWRG-------  114
Annona_cherimola_E_GH77_DPE2_C0L7E0_2                     LVQEDCMV---VQFKISC-PG----INEKASVCVLGDSIQLGLWKAQDALKLN-YI--------------GEFLWQGEC---TLLRA--------------E-L-PIKYKYCQIDQ---AGN------------VSLENGPDRELTVDATSKS---IPRYIFLSDGTFR-KT-PWRG-------  115
Hordeum_vulgare_E_GH77_DPE2_F2DIF3_2                      SLDPEDTV---VQFIISC-PR----LASGSTVVVTGSNPAVGNWKAQDGLKLN-YV--------------GDSIWKANC---VLRKS--------------E-F-PVKYKYCQVSE---AGV------------TSLEFGPNREVDVDLSSPK---PSRYVLLSDGSLR-EA-PWRG-------  115
Oryza_sativa_E_GH77_DPE2_Q69Q02_2                         SLEPEDIV---VQFIVSC-PR----LGAGSTVVVTGSNPQLGRWQTQDGLKLN-YV--------------GDSIWKANC---LLRKS--------------E-F-PIKYKYCKISE---AGV------------SSLEFGPNREADVDLSSPK---PSRYIVLSDGALR-ES-PWRG-------  115
Sorghum_bicolor_E_GH77_DPE2_C5X4T9_2                      SLDPEDIV---IQFVISC-PR----LVSGSTVIVTGSNPQLGRWQTQDGLKLS-YV--------------GDSLWKASC---ALRKS--------------E-F-PVKYKYCQISQ---AGN------------SSLELGPNREVNVDLSSPK---QSRYIVLSDGALR-DA-PWRG-------  115
Physcomitrella_patens_E_GH77_DPE2_A9TKS8_2                PLNPSAVV---VQFKIAN-SV----LHPDQAVFVIGSSSELGQWNEAAIALS-NT--------------VGYSWECLV---SISRT--------------E-F-PVSYKYILKNS---SGV------------VTYENDENRVLSLSLSAKK---SSSVLIAADGSFR-SK-PWRG-------  115
Flavobacteriaceae_bacterium_B_GH77_DPE2_C6X0I0_2          ---VSVLKKHTHLFRLEA-PI----YQKNWQIVMLGDHDALGNWEYFKAVPMA-QT--------------DFGVWEAAL---ELPA---------------N-Q-IIHYKYGLRNSD-TGEV------------FDIEYGENRWATPNTEKN------VLQIKADHYFRFKA-----------  110
Elizabethkingia_anophelis_B_GH77_DPE2_H0KPQ2_2            ---KIKPEQKESHLFTVQA-PL----YNKDEKIVLLGSAPELGNWNLNHYIEML-QT--------------DTITWEVFV---DLSD---------------N-I-YVEYKYAILNTK-TREI------------V-YENGPNRKVRTASNTE------TLNVLHDHYFK---------------  107
Ornithobacterium_rhinotracheale_B_GH77_DPE2_I4A298_2      ---TRALKKNTHRFEITF-PL----FDPNERLVILGDADGLGFWKEENAIFLN-PQ--------------GDGKWSIDV---DLSAE--------------N-N-YSAYKYAVYNTE-NQQI------------SYYENGENRVWAPNQSDE------DFVIVSDNNFRQ------------  109
Niastella_koreensis_B_GH77_DPE2_G8TPR9_2                  ---AKAVKNATHYFKVKA-PL----LEKNEVVCLLGSNKALADWDQEEPVLLH-RE--------------NNWWAVKL---HLPKE--------------S-F-PIQYKYGVYNIK-SNSF------------VRFETGENRWLHGDALVN------TLTILHDGFVHLPNTTWRG-------  115
Paludibacter_propionicigenes_B_GH77_DPE2_E4T2V1_2         ---LKQADTYTHLFKVKM-PV----LKNNEAICLIGDTCRALGSWSTNKPVILS-RE--------------KNNWTVGV---DLSNE--------------K-S-DVHYKYGDTKIDE-DHQF-----------RYFETGADRTAPIAKSNK------SLVILSDGFTRFPNQSWKG-------  116
Branchiostoma_floridae_E_GPDP5_C3Y330                      -------MTSQVTFRVKV--A----VTDRHGVVVVGDCPSLGGWDPHKAVELQ-RE--------------ENDVWSCTV---PLPEG--------------T--PVRYRYMVCDIR-QDKT---QK---KLMIIKWETYRQPRTFTPNGKSC--------AADGGSFGS---------YDG----  110
Homo_sapiens_E_GPDP5_Q9NPB8                                ------MTPSQVAFEIRG--T----LLPGEVFAICGSCDALGNWNPQNAVALL-PEN--------DTGESMLWKATI---VLSRG--------------V---SVQYRYFKGYFL-EPKTIGGPC---QVIVHKWETHLQPRSITPLESEI--------IIDDGQFGI------------------  115
Chondrus_crispus_E_LAF_R7QEI4_2                           ---SEQQEAVPVKFRIKY--R----PDEGELVYLLGNIPELGAWKKVHSPLM---------APAKN--GDGIFEVTMTIPRDAEY---------------E---EFEYKFVFIRRPD-G-------T------RRWEEGENRIARPFD-KAA-----P--REKDGDGAV-L--WDDRWEK----  116
Succinatimonas_hippei_B_GH77_DPE2_E8LIB5_2                -VSPANTLKQSLNLR-AW-CH----LDNNSALFITGDCKALGNWDPQKGVELV-QI--------------MPNPFSASL---DKDDL--------------P-S-YFEYKFAAINKD--NGE------------IFWEIGANRNASLQKGICEYPPES--------EVK-LY-PFSH-------  112
Cyanidioschyzon_merolae_E_LAF_M1UXX5_1                    ---RSVTPTIPTLFRVYC--H----TEFGDAVVAAGSHDKLGNWEPAKALRLR-H-Q----CQVDTP-FRDCWEGEV---DLVPE--------------S---SFEFKFVRLIGG-D--------------PQRALWETGPNRRAVIQRNSK-------------DGCLIE----VEWER----  112
Chondrus_crispus_E_LAF_R7QEI4_1                           -----------MQFRIR--S----AAYGDRLVLLGSTPALGKWDLNRAVP----------------CAWTS-GDVWVASHVARPFLHAP-------------S---RFEFKFVIIQKD-G-------P------PIWEPGPNHIAHIPVPHAL-----P--L------------V-ISIDWAR---  100
Arabidopsis_thaliana_E_GWD3_Q6ZY51                        ---KKDGSGTKVRLNVRLDHQ----VNFGDHVAMFGSAKEIGSWKKKSPLNW--------------------SENGWVCEL---ELDGG--------------Q---VLEYKFVIVKND-G--------S------LSWESGGNRVLKVPNSG-------------NFS-----VVCHWDA----  101
Oryza_sativa_E_GWD3_Q2QTC2                                ---RDSSKQPLVHLQVCLEHQ----VKFGEHVGIIGSTKELGSWEEQVELEW--------------------TTNGWVCQL---LVPGE--------------T---LVEFKFVIFLVG-G-------KD------KIWEDGNNRVVELPKDG-------------KFD-----IVCHWNR----  102
Homo_sapiens_E_GEN1_O95210                                ----AGSQQVSVRFQVYQ-VT----STDVQFIAVTGDHECLGRWNTYIPL---------------HYN--KDGFWSHSI---FLPAD--------------T---VVEWKFVLVENG-G-------V------TRWEECSNRFLETGHE---------------------KV-----VHAWWGIH---  100
Homo_sapiens_E_LAF_O95278                                 ---------MRFRFGVVV--P-PAVAGARPELLVVGSRPELGRWEPRGAVRLR-PAGTAAGDGALALQ--EPGLWLGEV---ELAAEEAAQDGAEPGRVD---TFWYKFLKREPG-G-------E------LSWEGNGPHHDRCCTYNEN---------NLVDGVYCL------------------  124
Nematostella_vectensis_E_LAF_A7SVW9                       -------MSITVKFSVVT--F----IDRETTVSVLGSVPELGSWNQKNRIELT-QSDSEF---R-LGPGLEPSFWSKDV---EMSFN-----------GKS---ELRFEYKFVSIK-NGN-------------FIWEGEGPCHNRKEVLTLNDKTASE-LLVNGVYIL----PVGIWIE----  125
Thermococcus_sp_B1001_A_GH13_2_CGT_Q9UWN2                 ---YLTNKQIPAIFEVRNTQGTNLETQVGEFLWLTGSVPELSYWSPETIKA-----V-----GPMLCP--GMPDWFVVA---SVPAD---------------T---YIEFKFLKAPLG-G-------T------GIWEVGSNHAYLTPSSG------------------IGEVSV--EANR-----  111
Geobacillus_stearothermophilus_B_GH13_2_MGA_P19531        ---ILSGTQTSVVFTVKSAPP----TNLGDKIYLTGNIPELGNWSTDTSGAVN-NAQ-----GPLLAP--NYPDWFYVF---SVPAG---------------K---TIQFKFFIKRAD-G-------T------IQWENGSNHVATTPTGA------------------TGNIT-----VTWQN----  111
Bacillus_circulans_B_GH13_2_CGT_P43379                    ---VLGSDQVSVRFVVNN-AT----TALGQNVYLTGSVSELGNWDPAKAIGP----M-----YNQVVY--QYPNWYYDV---SVPAG---------------K---TIEFKFLK-KQG-S-------T------VTWEGGSNHTFTAPSSG------------------TATIN-----VNWQP----  106
Microbulbifer_thermotolerans_B_GH13_2_M3H_A0A0A0Q4S7      ---VLSDDKNQVIFK-VN-AT----TSWGENIYIVGDIPELGSWDTSKAVG----------------PFHTP--NYPEWFLPV---SVPKN---------------T---TIQYKFIKKDSS-G-------N------VTWEGGNNRSITSPSSS------------------TGTLDT--SVLTWQ----  105
Thermoanaerobacterium_thermosulfurogenes_B_GH14_BAMY_P19584 ---LTPNGTIPVTFTINN-AT----TYYGQNVYIVGSTSDLGNWNTTYARG----------------PASCP--NYPTWTITL---NLLPG---------------E---QIQFKAVKIDSS-G-------N------VTWEGGSNHTYTVPTSG------------------TGSVT-----ITWQN----  104
Coralococcus_sp_EGB_B_GH13_6_M6H_A0A076EBZ6               ---PPTGTTANVEFVCNN-GT----TVMGQNVYVTGSVAELDTWSPTTTKI---------------LSPT---AMPTWRGTY---ALPAN---------------T---TVQWKCLKRDGS-G-------N------VVWQGGSDNTLTTPAAG------------------GSTTAT--ASF------  102
Pseudomonas_stutzeri_B_GH13_M4H_P13507                    ---GEPGALVSVSFRCDN-GA----TQMGDSVYAVGNVSQLGNWSPAAALR-------------LTDTS--GYPTWKGSI---ALPAG---------------Q---NEEWKCLIRNEA-N-------A----TQVRQWQGGANNSLTPSE-G-------------------ATTVGR----L-------  103
Arthrobacter_globiformis_B_GH31_6AGT_Q6BD65               ---TLGEESVWATFSCEN-AT----TTFGQSVYVVGNVPQLGNWSPADAVK-------------LEPS--AYPTWTGVV---RNLPPS---------------S---TVEWKCIKRQEA-G-------L---PNTADAWEPGGNNILSTPPSG------------------SAGITT----GAF------  107
Bacillus_circulans_B_GH13_ICGT_A0P8W9                     ----GEPQADSVTFRVSVPGS----TPANAPVYLTGS---FNSWNAADTALL-T--------------RGS--DGIYSITL---NLPAG---------------T---AVTYKLTRG-SWATVET---T------SSGADITNRT-LTPAGGAQ----------------TVTI----SVQRWKDQ---  107
Bacillus_circulans_B_GH119_AAMY_A0P8X0                    ---TGEPQADSVTFRVSVPGS----TPANAPVYLTGS---FNSWNAADPAYLL-T--------------RGS--DGIYSITL---NLPAG---------------S---AVTYKLTRG-SWATVET---A------SSGADITNRT-LTPAGGAQ----------------TVTL----TVQRWKDQ---  108
Thermococcus_kodakarensis_A_CE1_HYPO_Q5JF12               ---KTESREITVTFIVSVPGY----TPEDDAVYIAGD---FNGWNPGDENYRL-T--------------KLP--DGRWAINL---TFPYG---------------K---VIEFKFTRG-SWETVEK---G------PNGEEIPNRQ-FVFKD-SG-----------------TYEF----TVYHWRD----  106
Kosmotoga_olearia_B_GH57_APUL_C5CEB0_1                   ---PSPKGTTLVTFNVTLPDN----TPASDRIYLAGN---FNGWDPSSPKFIM-E--------------RTG--N-TAVLVV---EMPIG---------------D---EIEYKYTRG-GWNRVEK---ATVTE-NL------PGEEISNRK-ATVTE-NL-------------VLND----VVASWAD----  105
Kosmotoga_olearia_B_GH57_APUL_C5CEB0_3                   ---VPTGTPARVIFKVKLPWN----TPPFDDIYITGT---FNGWKTGDPDTIL-E--------------RKG--E-TATITL---DAKVG---------------D---EIRYKYNRG-SWETVEK---D------KEGNEIPNRV-VIVDKTEV-----------------IVED----TIESWID----  106
Kosmotoga_olearia_B_GH57_APUL_C5CEB0_2                   ---AQPGEEVPVTFVVTVPEN----TPE-GPIYMVGS---FNEWNPGDPAYEM-K--------------RTG--N-VAKITL---KLPAG---------------S---TIEYKYTRG-DWGTVEK---G------PEGEEIPNRT-VKVSP-GL-----------------TVED----VVASWAD----  104
Geobacillus_thermoleovorans_B_GH13_39_APUL_I1WWV6         ---TPDIVMVKVIFKVKAPDY----TPLDARITIPNS---LNGWNTGA--WEM-S--------------RNGAVTPDWQFTT---EVQEG---------------E---TITYKYVKGGSWDQEGL---A------DHTREDDNDDDVSYYG-YG------------------T-----------------  98
Bacillus_sp_XAL601_B_GH13_39_APUL_Q45643                 ---TPDIVMVQVTFKVKAPSY----TPLDTRITIPNS---INGWNTGA--WEM-T--------------RGGAVTPDWEFTT---ELQEG---------------E---TITYKYVKGGSWDQEGL---A------DHTRDDQTDDDVSYYG-YG------------------A-----------------  98
Streptomyces_griseus_B_GH13_32                            ---GPGTGQTSASFHVNA--T----TAWGENIYVTGDQAALGNWDPARALKL--------------DPA--AYPVWKLDV--P-LAAG----------------T---PFQYKYLRKDAA-G-------K------AVWESGANRTATVGTTG----------------------ALT--LNDTWRG----  102
Pseudomonas_sp_KO8940_B_GH13_M5H_Q52516                  ---AAGAAVLSLTFNETA--D----TVWGQNLFVVGNVGALGNWAPAAGAAM---T----WISGSG--STGQWRATV---Q-LPAD---------------Q---PVQYKYVKKDGA-G-------N------VVWESGGNRVVTTPAPG---------------------A--TIA--VNDSWK----  106
Apsergillus_kawachii_E_GH13_1_AAMY_O13296                ---TATSTTLPITFEELV--T----TTYGEEVYLSGSISQLGEWHTSDAVKL----S-----ADDYTS--SNPEWSVTV--S-LPVG---------------T---TFEYKFIKVDEG-G-------S------VTWESDPNREYTVPECG------------------SGSGET--VVDTWR----  108
*Aspergillus_niger_E_GH15_GAMY_P69328                     ---CTTPTAVAVTFDLTA--T----TTYGENIYLVGSISQLGDWETSDGIAL----S-----ADKYTS--SDPLWYVTV--T-LPAG---------------E---SFEYKFIRIESD-D-------S------VEWESDPNREYTVPQAC------------------GTSTAT--VTDTWR----  108
Neurospora_crassa_E_AA13_LPMO_Q7SCE9                      ---CTPAATVAVTFNHLA--S----TSYGESIKIVGSISQLGSWSASSGVAL----S-----ASQYTT--SNPLWTATV--S-LPAG---------------T---KFEYKFVKVSSE-G-------SA-----VTWESDPNRSYTVPQSC------------------AESVAV--ESSWK----  108
Aspergillus_nidulans_E_AA13_LPMO_Q5B1W7                   ---CASAISIPVTFNALV--T----TTYGENVYLAGSISQLGSWSTSSAVAL----S-----ASKYSS--SSPLWTVTV--D-LPVG---------------A---TFEYKYIKKESD-G-------S------IVWESGPNRSYTVPTGC------------------SGTTAT--ESGAWR----  108
Bacillus_cereus_B_GH14_BAMY_P36924                        ---LLGVTPVMQTIVVKN-VP----TTIGDTVYITGNRAELGSWDTKQYPIQ-------------LYYDS--HSNDWRGNV---VLPAE---------------R---NIEFKAFIKSKD-G-------T------VKSWQTIQQSWNPVPLKT------------------TSHT-------SSW-----  103
Bacillus_megaterium_B_GH14_BAMY_Q9RM92                    ---TLGVTPLAQTVVVKN-AP----TALGETVYIVGDRAELGQWDTSIYPIK------------LTYNS--STADWRGTV---HFPAS---------------Q---NVQFKAIVKRAD-G-------S------LKAWQPSQQYWSV-PSTT------------------TTYT-------DNW-----  102
Hormoconis_resinae_E_GH15_GAMY_Q03045                     ---VTSSCQVSITFNVKA--T----TYYGENLYVIGNSSDLGAWNIADAYPL----S-----ASAYTQ--DRPLWSAAI--P-LNAG---------------E---VISYQYVRQEDC-D-------QP-----YIYE-TVNRTLTVPACG------------------G-AAVT--TDDAWMG----  108
Penicillium_oxalicum_E_GH15_GAMY_S7ZIW0                  ---IPAKDIVPITFYLIE--N----TYYGENVFMSGNITALGNWDAKKGFPL----T-----ANLYTQ--DQNLWFASV--E-FIPAG---------------T---PFEYKYVKVEPN-G-------D------ITWEKGPNRVFVAPTGC------------------PVQP-H--SNDVWQS----  109
Hordeum_vulgare_E_GH77_DPE2_F2DIF3_1                      ----MGKNTVTVIFKLPY--Y----TQWGQSLVIAGSAPALGSWSVKQGLSLS-PVH---------QG--NALVWCGQV---SVAAG---------------F---TCEYSYHVVDDH-KNVL-------------RWESGEKKKLVL---PEG-----------------VQDGEVVEVRDWW-----  108
Sorghum_bicolor_E_GH77_DPE2_C5X4T9_1                      ---KKSLNSVTLLFKLPY--Y----TQWGQSLLIAGSEPALGSWNVKQGVPLS-PVH---------QD--SELIWCGSV---SVVAG---------------F---TCEYKYYVVDDN-KNVL-------------RWEAGEKKKLVL---PEG-----------------VKEGDVIEIRDWWT----  109
Oryza_sativa_E_GH77_DPE2_Q69Q02_1                         ---KKSLNTVTLVFKLPY--Y----TQWGQSLLIAGSEPALGSWNVKQGISLS-PVH---------QG--NELIWSGRV---SVATG---------------F---TCQYNYYVVDDN-KNVL-------------RSESGEKKKLVL---PEG-----------------VQDGDVVEIRDWWQ----  109
Annona_cherimola_E_GH77_DPE2_C0L7E0_1                     ---KKSLKSVSLSFRLPY--Y----TQWGQSILVSGSEPVLGSWNVKHGLLLC-PTH---------QG--GELVWSGRI---SVPTA---------------F---GCEYNYYVLVDDD-KNIL-------------RWEAGQKRRLIL---PEG-----------------IQEGAVVELHDLWQT----  110
Populus_trichocarpa_E_GH77_DPE2_B9IHJ8_1                  ---TKTAKSVNVSFRLPY--Y----TQWGQSLLVCGSERVLGSWDVKKGLLLS-PVH---------QG--EELIWSGSI---SVPSE---------------F---SGEYSYYVVDDK-KSVL-------------RWEMGKKRKLVL---PEG-----------------INGGEHVELHDLWQA----  110
Linum_tenue_E_GH77_CAI0439830_1                           ---SKSSKSMRVSFRIPY--Y----TNWGQSLVVCGSDPVLGSWNVKKGLLLK-PVH---------QD--DELIWSGSI---SVPAE---------------F---SCEYSYYVVDDE-RNVL-------------RWEMGNKRALFS---PQG-----------------INGGDIVELHDLWQT----  110
Ricinus_communis_E_GH77_DPE2_B9SCF0_1                     ---TESVKSVNLSFRIPY--Y----TQWGQSLLVCGSKPILGSWNVKKGLLLS-PVH---------EG--EELIWYGTV---SIPSA---------------F---SCEYSYYVVDDE-RNVL-------------RWEMGKKRKLLL---PEC-----------------VNDGETAELHDLWQS----  110
Arabidopsis_thaliana_E_GH77_DPE2_Q8RXD9_1                 ---KSSKPMVSISFWIPY--F----THWGESLLVCGSAPGLGSGNVKKGLLLK-PSQ---------QD--DQLIWSGSV---SVPPG---------------F---SSDYCYYVVDDS-KSVL-------------RSEFGMKRKLVV---PET-----------------LTGGESVHLRDLWQS----  110
*Solanum_tuberosum_E_GH77_DPE2_Q6R608_1                   -MVNSGLKSRKVSFRIPY--Y----TQWGQNLLICGSDRLLGSWNVKKGLLLK-PSH---------QG--EVLVWSGSI---PVPPG---------------Y---QSEYSYYVVDDE-RNIL-------------RWEVGKKRKLLL---PDG-----------------LQDGQSLELRDLWQT----  112
Physcomitrella_patens_E_GH77_DPE2_A9TKS8_1                ---LRRLKRLNILFKLPY--N----TQWGQNLVIIGSDALLGAWAVVEKGQLM-PRQ---------EG--DVLVWEVSI---SVSER---------------F---ETEYNYYVVDDR-FRGL-------------RRESGARRVLSL---PEG-----------------LSNGATVELHDLWQD----  110
Selaginella_moellendorffii_E_GH77_DPE2_D8S7D7_1          -ERAAEPRRVSLRFRIPY--F----TQWGQNLIVTGSHSSLGSWNLHQGLAMV-PHH---------DEELGELVWTAQL---AVREG---------------F---QARYSYCVVDDK-RNAV-------------RREAGIPRSFVL---PDG-----------------IPNGSIVDLYDLWQR----  114
Micromonas_sp_RCC299_E_GH77_DPE2_C1FJ00_1                 ---MSVIPKTTVFHFRIKY--W----TEWGQSLVVCGNDPRLGSWDVRKGVPLR-CQH---------LGE-DQLLWTGSV---TGHFV---------------G---SFDYRYAVVDRD-MNVV-------------RWDAIVRHAHFAPAMPEG-----------------RGGGSDDNSSEAGSS----  114
Chlamydomonas_reinhardtii_E_GH77_DPE2_A8JEI0_1           -MSQHPGISVTLHFSVAY--P----TQWGQCILLAGSGALLGGLEWSKARQLS-CTN---------DK--DLIVWEATI---VLPWK---------------P---SYTYKYALDD-------TSGPSVHGNWIIEKEDAAGERTVWL---PEG-----------------LQGGLEVEVVDSWVD----  119
Volvox_carteri_E_GH77_DPE2_D8UDU0_1                       ---PWQQASVTLHFSVSY--P----TQWGQSILLSGSDALLGNWSQSQARQMS-CHH---------EK--DVLVWEATI---LLPWK---------------P---YYTYKYALARQK-DDEVAPGANVHGNWIIEKEDAAGERTVWL---PEG-----------------LQGGDLVEVCDTWVA----  123
Paludibacter_propionicigenes_B_GH77_DPE2_E4T2V1_1         ---------MRIHFNIPF--F----TRWGQRLLVTGNIPELGDNDKTKALPLN-FQY---------KE----DWSGEI---EIPNTE-------------SF---ELIYKYILFNDE-TGQF-------------VEEWGDDRVINI-----Q--------------------PSKTDTIYCFDKWNA----  101
Nostoc_sp_PC9229_B_GH13_2_CGT_Q8RMG0                     ERIKGQTI---VRAQLNG-VQ----TQPGETIVVIGDCPELGNWDISKAYPLE-YI--------------NTNTWFAEI---PFNES-------------AGK-LIAYKYALWRE--GQS----------------PLRENLVN-------------------RRWVIAKEGTV-----KWRDTWASGRES  109
Cyanidioschyzon_merolae_E_LAF_M1UXX5_2                    ---------TRVLFSIYY---P----TKEKGQHLCVTGDLPEIGRWVEPGVPMA-LSTTEE--K-LETGGKGRKWSLTV---SVPST-------------VG---KFAYRYVLVDN-RQQ-------------TIWEREPNRYATLERA---VNGRLE---C---FDA-----------------  107
Chondrus_crispus_E_LAF_R7QEI4_3                           ----------IEFSIYY---A----TKTNQVMYVTGDPAQIGAWFKPGTPRMT-LGPV-E---V-LETDVGRKWYLSV---WTDPK-------------QK---PFSYRYIIINEK-TKQ-------------ELWEREPNRRGEFEAA---DPVVN---S----VRI-----------------  103
Barnesiella_intestinihominis_B_GH77_DPE2_K0XAQ2_1         ---------MKITFEIYY--H----TRWGESLFLSGDIDELGANEDNRAVMMD-YQG--------------DG----LWKYTM---ELPASA-------------K---KFHYEYLVKS---GEGI-------------RREWGSPHSFSP-----------------KN-AAEYRLVDRWRD----  97
Parabacteroides_distasonis_B_GH77_DPE2_A6L7Y4_1           ---------MKVSFNINF--H----TVWGQKLCVVGSIPELGSWEPALAKEMN-YSG--------------DG----LWKLEL---DLPPDI-------------K---DIEYRYFLSVN-DKQI-------------FEEWEKNHRIVL-----D-------------------GQ-SDSYILYDYWQI----  98
Dysgonomonas_mossii_B_GH77_DPE2_F8WZF9_1                  ----------KIKLHIDY--H----TVWGQNIYVCGSCPELGNWDEKKALKMT-CIS--------------SS----LWIVDF---E--TND--------------T---SIEYRYIVKEN--KVVT-------------AQEWGDPHTLLL-----D-------------------AS---KTFDVLDSWRG----  94
Trichomonas_vaginalis_E_GH77_DPE2_A2D7I8_1               -MNFISSNGVIVHFHVRV-----STTFGQEVFVSGNIPELGNWDSEKSIKM------------HFEQ--NLDYWTVDVELQKSKDP----------------T---IVEYKYIISNGD-N--------------KQWEPEQNHKLVLGEIN------------------ENCIINIEDHFHWRD----  112
Dictyostelium_discoideum_E_GH77_DPE2_Q54PW3_1             -------MANFIRFKV------NYFTRLGQELYVSGSTEPLGNWDESKATK-------------LTYC--DNGDWETLI---QFPASN---------------N---KVDYKYFVMDTN-G-------E------KIWESGSNRTFNLNDLN-----------------SNYIHE---FRDTYQS----  102
                                                                                                   1                  2  2                    1                   1
```
